# Rare central nervous system tumors in adults: a population-based study of ependymomas, pilocytic astrocytomas, medulloblastomas, and intracranial germ cell tumors

**DOI:** 10.1093/noajnl/vdac062

**Published:** 2022-04-22

**Authors:** Vincent K Y Ho, Anja (J) M M Gijtenbeek, Michiel Wagemakers, Walter Taal, Myra E van Linde, Annemarie T Swaak-Kragten, Erkan Kurt, Hiske L van der Weide, Pieter Wesseling, Filip Y de Vos, Jacoline E C Bromberg

**Affiliations:** Department of Research & Development, Netherlands Comprehensive Cancer Organization (IKNL), Utrecht, The Netherlands; Department of Neurology, Radboud University Medical Center, Nijmegen, The Netherlands; Department of Neurosurgery, University Medical Center Groningen, Groningen, The Netherlands; Department of Neurology/Brain Tumor Center, Erasmus MC Cancer Institute, Rotterdam, The Netherlands; Department of Medical Oncology, Amsterdam University Medical Center, Amsterdam, The Netherlands; Department of Radiation Oncology, Erasmus MC Cancer Institute, Rotterdam, The Netherlands; Department of Neurosurgery, Radboud University Medical Center, Nijmegen, The Netherlands; Department of Radiotherapy, University Medical Center Groningen, Groningen, The Netherlands; Department of Pathology, Amsterdam University Medical Center, Amsterdam, The Netherlands; Department of Pathology, Princess Máxima Center for Pediatric Oncology, Utrecht, The Netherlands; Department of Medical Oncology, University Medical Center Utrecht, Utrecht, The Netherlands; Department of Neurology/Brain Tumor Center, Erasmus MC Cancer Institute, Rotterdam, The Netherlands

**Keywords:** ependymoma, epidemiology, intracranial germ cell tumor, medulloblastoma, pilocytic astrocytoma

## Abstract

**Background:**

Ependymomas, pilocytic astrocytomas, medulloblastomas, and intracranial germ cell tumors occur relative frequently in children, but are rare central nervous system (CNS) tumors in adults. In this population-based survey, we established incidence, treatment, and survival patterns for these tumors diagnosed in adult patients (≥18 years) over a 30-year period (1989–2018).

**Methods:**

Data on 1384 ependymomas, 454 pilocytic astrocytomas, 205 medulloblastomas, and 112 intracranial germ cell tumors were obtained from the Netherlands Cancer Registry (NCR) on the basis of a histopathological diagnosis. For each tumor type, age-standardized incidence rates and estimated annual percentage change were calculated. Trends in incidence and main treatment modalities were reported per 5-year periods. Overall survival was calculated using the Kaplan–Meier method, and relative survival rates were estimated using the Pohar-Perme estimator.

**Results:**

Incidence and survival rates remained generally stable for pilocytic astrocytomas, medulloblastomas, and germ cell tumors. Increasing incidence was observed for spinal ependymomas, mostly for myxopapillary ependymomas, and survival improved over time for grade II ependymomas (*P* < .01). Treatment patterns varied over time with shifting roles for surgery in ependymomas and for chemotherapy and radiation in medulloblastomas and germinomas.

**Conclusions:**

The study provides baseline information for highly needed national and international standard treatment protocols, and thus for further improving patient outcomes in these rare CNS tumors.

**Key Points**

- The incidence of spinal ependymomas, mostly myxopapillary ependymomas, has increased significantly in adult patients over 30 years.
- Survival has improved significantly for adult patients with grade II ependymomas.

**Importance of the Study**

Due to their rarity, population-based information on adult patients with ependymomas, pilocytic astrocytomas, medulloblastomas, or intracranial germ cell tumors are scarce. This comprehensive survey reports on long-term patterns of incidence, treatment, and survival for these tumors diagnosed in an unselected patient population, and stresses the need for standard treatment protocols.

Most primary neoplasms that frequently affect the central nervous system (CNS) in children are rare in adulthood. They may nevertheless require due consideration since they are occasionally encountered by neuro-oncology clinics treating adult patients. These tumors include ependymomas, pilocytic astrocytomas, medulloblastomas, and intracranial germ cell tumors.

Given their rarity, treatment choices in adult patients are seldom substantiated by outcomes of large series, and consensus regarding their optimal management is generally lacking. Instead, treatment plans for adult patients are frequently extrapolated from available pediatric protocols. Population-based data may aid clinical practice not only by showing changes in incidence rates over time but also by generating information on survival, treatment, and possible treatment effects in unselected populations.

The Rare Cancer Working Group of the Dutch Neuro-Oncology Society (LWNO) has specifically aimed to investigate and improve clinical management of ependymomas, pilocytic astrocytomas, medulloblastomas, and intracranial germ cell tumors in adults. In the Netherlands, pediatric oncology including neuro-oncology has been centralized for several decades, with children who have these tumors being treated in university hospitals, and, as of 2018, in a single national pediatric oncology center. In contrast, their variants in adults are still treated in nearly every neuro-oncology center.

Ependymomas are neuroepithelial tumors considered to originate from (precursors of) ependymal cells covering the walls of the ventricular system (including the central canal in the spinal cord).^1–3^ Compared to pediatric patients, ependymomas in adults are more often found in the spinal cord, and are less often grade III.^4,5^ Overall, prognosis appears more favorable for younger adults compared to children and the elderly.^6^

Pilocytic astrocytomas (including their variant pilomyxoid astrocytomas) are considered benign, classified by the WHO as grade I, with the supratentorial compartment as foremost location in adults.^7^ With a significant proportion following an aggressive clinical course, prognosis for adult patients appears less favorable than for children, likely because of different molecular characteristics.^8,9^

Medulloblastomas are grade IV embryonal tumors of the CNS. Although they constitute a relatively common brain malignancy in childhood, they are only very rarely diagnosed in adults, accounting for 1%–2% of primary adult brain tumors.^10^ As in children, leptomeningeal spread is associated with a poorer prognosis, which may concern tumor cells in the cerebrospinal fluid as well as localized metastatic disease visualized on MRI.^11^

Intracranial germ cell tumors comprise a very heterogeneous group of neoplasms with 2 main categories: germinomas and nongerminomatous tumors. Germinoma is the most common subtype in the CNS, accounting for approximately two thirds of germ cell tumors.^12,13^ Prognosis is highly variable and depends on tumor histology and dissemination, with (localized) germinomas and mature teratomas exhibiting favorable outcomes given optimal treatment, while prognosis for other germ cell tumors is far worse.^14^ Due to their scarcity in adults, no comparisons can be made with the pediatric patient population.

This population-based survey was set up in line with the objectives of the Rare Cancer Working Group of the LWNO. To this end, data were extracted from a comprehensive national cancer registry and analyzed to report on the epidemiology and treatment patterns of ependymomas, pilocytic astrocytomas, medulloblastomas, and intracranial germ cell tumors diagnosed in adults over a 30-year period.

## Materials and Methods

### Case Selection

For our study, we derived electronic patient records from the Netherlands Cancer Registry (NCR), which nowadays covers over 17 million inhabitants (14.8 million in 1989) and is hosted by the Netherlands Comprehensive Cancer Organization (IKNL). The NCR has an estimated coverage of 90%–95% of incident malignancies in The Netherlands from 1989 onward,^15^ with CNS tumors of nonmalignant (benign and uncertain) behavior having been systematically included since 1999. Newly diagnosed patients are added to the registry through reports on histological, cytological, and autopsy examinations by all pathology laboratories in the Netherlands. Following notification, data managers of IKNL collect additional information from hospital records on patient and tumor characteristics, diagnostics, and therapeutic interventions that are part of the primary treatment plan. Follow-up information on vital status is obtained on a yearly basis through linkage with the Municipal Personal Records Database (Gemeentelijke Basisadministratie, GBA). Vital status for this study was updated until January 31, 2021. The study design, data abstraction process, and storage protocols were approved by the national supervisory committee of the NCR.

From the NCR database, we selected adult patients (≥18 years of age at time of diagnosis) with a histological diagnosis of ependymoma, pilocytic astrocytoma, medulloblastoma, or intracranial germ cell tumor diagnosed during the period 1989–2018. Tumors were identified on the basis of their International Classification of Diseases for Oncology (ICD-O) topography and histology codes, and clustered according to the 2016 WHO classification scheme (see Supplementary Table A; for translation into the 2021 classification, see Supplementary Table B). Because of the extensive time period studied, diagnoses were based on histology only. We excluded cases diagnosed postmortem.

### Statistical Analyses

For overall incidence, we calculated annual rates per 100 000 person-years with corresponding 95% confidence intervals (95% CIs) for each tumor type using the average annual population as provided by Statistics Netherlands (Centraal Bureau voor de Statistiek, CBS). The rates were age adjusted through standardization to the world standard population (World Standardized Rate, WSR), and tabulated by histology and by gender. We estimated trends in incidence by assessing the estimated annual percentage change (EAPC). As the registry should be considered incomplete for subependymoma, myxopapillary ependymoma, and pilocytic astrocytoma before 1999, rates for these tumor types as well as for the main groups of ependymomas were calculated over the period 1999–2018 only. In presenting time trends in incidence and treatment, the study period was divided in 5-year intervals, and estimates were averaged within each interval. Trends in treatment modalities were evaluated using logistic regression models. Overall survival (OS) was calculated from time of diagnosis by the Kaplan–Meier method, and we applied log-rank tests to compare survival rates between patient subgroups. In addition, net survival analyses were performed as an approximation of disease-specific survival. In the relative survival setting (RS), we calculated ratios of OS in patients to the expected survival in the general Dutch population by matching cases to annual life tables on age, gender, and calendar year (retrieved from CBS) using the Pohar-Perme estimator.^16^ Relative survival rates between subgroups were evaluated by applying a Poisson regression model. All statistical analyses were 2 sided, with a *P* value <.05 being considered significant. Analyses were performed using software package Stata version 17.0 (StataCorp).

## Results

Our query on the NCR database yielded information on 1384 ependymomas, 454 pilocytic astrocytomas, 205 medulloblastomas, and 112 germ cell tumors in adults (Table 1). Patients with ependymomas were somewhat older (median 48 years, with an interquartile range [IQR] of 37–59 years) than the other studied tumor types (median 29 years, IQR 22–41 years) and there was a general male predominance which was most explicit in patients with germ cell tumors (80%) and specifically germinomas (82%). Resection was the most prevalent treatment modality in all tumor types, with over 87% of all patients undergoing resection, with the, exception of patients with germ cell tumors, especially germinomas in whom only 28% of patients had a resection.

**Table 1.** Characteristics of adult patients with ependymoma, pilocytic astrocytoma, medulloblastoma, and intracranial germ cell tumors diagnosed in the Netherlands from 1989 to 2018

Tumor type (WHO 2016)	Total		Crude rate	WSR	Median age (interquartile range)	Male/ female	Resection	Radiotherapy^b^	Chemotherapy ^b^
	*n*	%			Years	%/%	%	%	%
Ependymoma^a^	1384	100.0%	0.42	0.27	48 (37–59)	59.5/40.5	92.5	24.2	0.6
Supratentorial ependymoma^a^	362	26.2	0.10	0.06	51 (38–63)	62.7/37.6	85.6	35.9	1.4
Infratentorial ependymoma^a^	243	17.6	0.08	0.05	53 (40–63)	64.6/35.4	91.8	31.7	0.4
Spinal ependymoma^a^	779	56.3	0.24	0.16	46 (35–57)	56.6/43.4	95.9	16.4	0.3
Subependymoma^a^	158	11.4%	0.06	0.03	54 (44–63)	74.1/25.9	89.9	0.6	—
Myxopapillary ependymoma^a^	283	20.4%	0.10	0.07	44 (32–56)	60.8/39.2	97.5	12.7	0.4
Papillary/clear cell/tanycytic ependymoma	819	59.2%	0.21	0.14	48 (38–59)	57.3/42.7	93.0	25.3	—
Anaplastic ependymoma	124	9.0%	0.03	0.03	49.5 (31–62.5)	53.2/46.8	80.6	73.4	5.6
Pilocytic astrocytoma	454	100.0%	0.13	0.14	31 (22–47)	57.9/42.1	82.6	9.7	1.1
Medulloblastoma	205	100.0%	0.05	0.05	28 (23–36)	62.0/38.0	89.8	88.8	26.3
Medulloblastoma, classic/NOS	155	75.6%	0.04	0.04	28 (23–37)	62.6/37.4	88.4	87.7	24.5
Desmoplastic/nodular medulloblastoma/with extensive nodularity/SHH-activated and TP53-wildtype	46	22.4%	0.01	0.01	27.5 (20–35)	60.9/39.1	93.5	91.3	30.4
Large cell/anaplastic medulloblastoma	4	2.0%	0.00	0.00	34.5 (26–39)	50.0/50.0	100.0	100.0	50.0
Intracranial germ cell tumor	112	100.0%	0.03	0.04	23 (20–31)	80.4/19.6	39.3	70.5	24.1
Germinoma	83	74.1%	0.02	0.03	23 (19–29)	81.9/18.1	27.7	85.5	19.3
Nongerminoma	29	25.9%	0.01	0.01	27 (20–45)	75.9/24.1	72.4	27.6	37.9
Embryonal carcinoma	2	1.8%	0.00	0.00	24.5 (22–27)	100.0/—	—	100.0	100.0
Yolk sac tumor	2	1.8%	0.00	0.00	25.5 (20–31)	100.0/—	—	50.0	100.0
Teratoma	10	8.9%	0.00	0.00	34 (20–61)	60.0/40.0	90.0	20.0	20.0
Teratoma with malignant transformation	10	8.9%	0.00	0.00	35 (23–45)	70.0/30.0	80.0	—	10.0
Mixed and other germ cell tumor	5	4.5%	0.00	0.00	20 (20–30)	100.0/—	80.0	60.0	80.0

NOS, not otherwise specified; WSR, World Standardized Rate.

^a^Incidence rates calculated over the period 1999–2018.

^b^As (part of) primary treatment.

### Ependymoma

**Figure 1.**
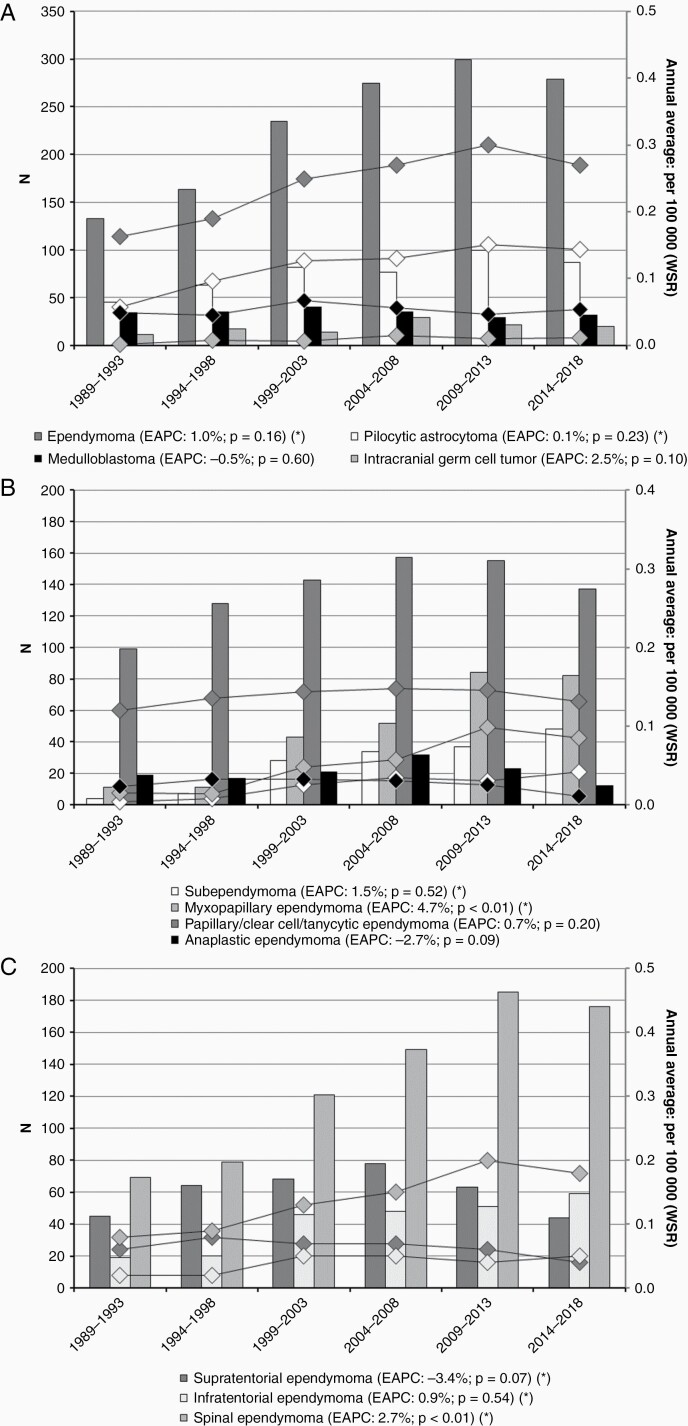
Crude numbers (bars, left axis) and annual averaged, age-adjusted incidence rates (lines, right axis) for adult ependymoma, pilocytic astrocytoma, medulloblastoma, and intracranial germ cell tumors diagnosed in the Netherlands from 1989 to 2018. (A) Incidence of adult ependymoma, pilocytic astrocytoma, medulloblastoma, and intracranial germ cell tumors. (B) Incidence of adult ependymoma subtypes. (C) Incidence of adult ependymoma by localization. *Estimated annual percentage change (EAPC) calculated over the period 1999–2018.

**Figure 2.**
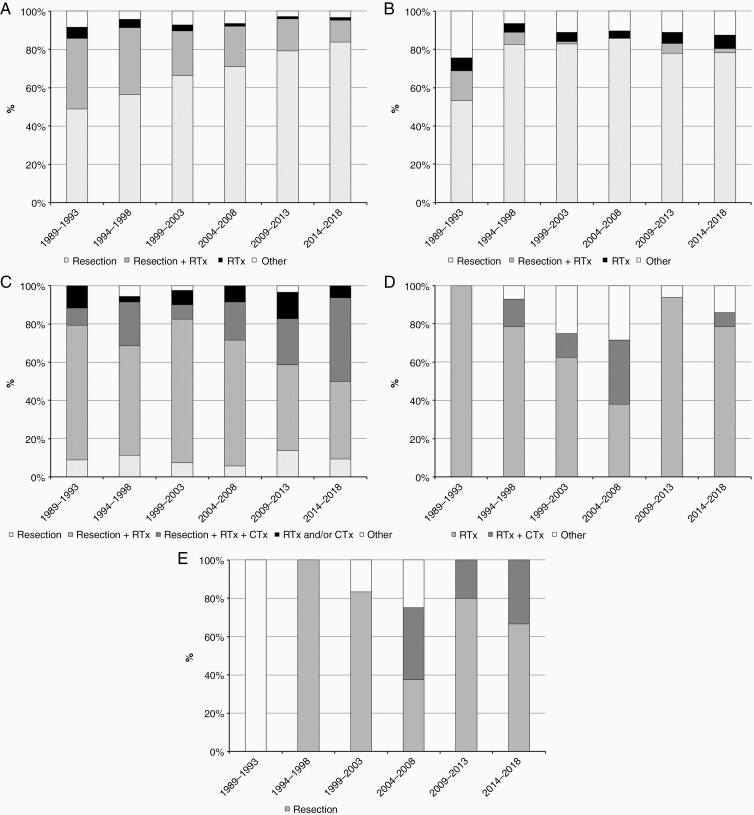
Trends in primary treatment of adult ependymoma, pilocytic astrocytoma, medulloblastoma, and intracranial germ cell tumors diagnosed in the Netherlands from 1989 to 2018. (A) Primary treatment of adult ependymoma. (B) Primary treatment of adult pilocytic astrocytoma. (C) Primary treatment of adult medulloblastoma. (D) Primary treatment of adult intracranial germinoma. (E) Primary treatment of adult intracranial nongerminoma.

The majority of the 1384 adults with an ependymoma were histologically diagnosed with a grade II ependymoma (59.2%), while to somewhat less than one-tenth of cases (9.0%) a grade III was assigned. Most ependymomas were located in the spine (56.3%; Table 1). Median age was highest among patients with a subependymoma (54 years, IQR 44–63 years), who also had the highest male predominance (74.1%). While the overall incidence of ependymoma remained stable from 1999 onward (EAPC 1.0%, *P* = .16; Figure 1A), the rate for myxopapillary ependymoma increased from 0.02 per 100 000 inhabitants in 1999–2003 to 0.09 in 2014–2018 (EAPC 4.7%, *P* < .01; Figure 1B), which coincided with a rise in spinal ependymal tumors (EAPC 2.7%, *P* < .01; Figure 1C). The proportions of subependymoma and myxopapillary ependymoma increased from 11.9% and 18.3% of the total number of incident ependymal tumors in 1999–2003 to 17.2% and 29.4% in 2014–2018, respectively, while the proportions of grades II and III tumors decreased from 60.9% and 8.9% to 49.1% and 4.3%, respectively. In the management of ependymoma in adults, the use of radiation therapy—as either adjuvant to surgical resection or as primary treatment—steadily decreased over time, from 26.4% in 1999–2003 to 12.9% in 2014–2018 (Figure 2A). This trend was observed for both brain (31.6%–19.4%) and spinal tumors (21.5%–9.1%), and mostly concerned grade I (11.3%–5.4%) and grade II tumors (27.3%–14.6%). Radiation therapy for grade III tumors slightly increased from 71.4% to 75.0%. Ependymomas located in the spine had a better prognosis than intracranial ependymomas, with a 5-year OS of 93.2% (95% CI: 91.2%–94.8%; Table 2) versus 67.6% (95% CI: 65.4%–69.8%; *P* < .01), and a 5-year RS rate of 95.8% (95% CI: 93.3%–97.3%) versus 70.1% (95% CI: 65.9%–73.8%; *P* < .01). Median survival was 12.6 years for supratentorial ependymoma and 15.4 years for infratentorial ependymoma, and was not reached for spinal ependymoma. Between 1999–2008 and 2009–2018, survival improved for all subtypes of ependymoma, but only significantly for grade II tumors (*P* < .01).

**Table 2.** Survival of adult patients with ependymoma, pilocytic astrocytoma, medulloblastoma, and intracranial germ cell tumors diagnosed in the Netherlands from 1989 to 2018

Tumor type (WHO 2016)	Five-year overall survival	95% CI	Median survival	95% CI	Five-year relative survival	95% CI
	%		Years		%	
Ependymoma	82.0	(79.9–84.0)	29.6	(24.4–)	84.5	(82.2–86.5)
Supratentorial ependymoma	64.0	(58.7–68.7)	12.6	(9.8–17.0)	66.0	(60.6–71.0)
Infratentorial ependymoma	73.2	(67.0–78.4)	15.4	(13.8–)	76.1	(69.6–81.5)*
Spinal ependymoma	93.2	(91.2–94.8)*	Not attained		95.8	(93.3–97.3)*
Grade I ependymoma	93.1	(90.2–95.1)	Not attained		95.8	(92.1–97.8)
1989–1998	87.5	(70–95.1)	25.1	(19.1–)	89.4	(68.8–96.7)
1999–2008	91.0	(85.4–94.6)	Not attained		93.6	(86.1–97.1)
2009–2018	95.4	(91.8–97.4)	Not attained		98.5	(90.6–99.8)
Grade II ependymoma	84.0	(81.3–86.3)	29.6	(24.4–)	86.6	(83.7–89)
1989–1998	81.0	(75.2–85.5)	28.9	(19.4–)	83.3	(77.1–87.9)
1999–2008	81.7	(76.8–85.6)	Not attained		84.1	(78.9–88.1)
2009–2018	88.7	(84.3–92)*	Not attained		91.7	(86.7–94.9)*
Grade III ependymoma	30.9	(23–39.2)	1.9	(1.3–3.1)	31.6	(23.3–40.1)
1989–1998	30.6	(16.6–45.7)	1.3	(0.6–3.9)	31.3	(16.4–47.5)
1999–2008	27.9	(16.6–40.4)	1.3	(0.9–3.3)	28.9	(17.2–41.5)
2009–2018	35.6	(20–51.5)	3.3	(1.6–5.4)	35.7	(19.7–52)
Pilocytic astrocytoma	83.1	(79.3–86.3)	Not attained		84.3	(80.3–87.5)
1989–1998	73.0	(63.5–80.4)	Not attained		74.2	(64.3–81.8)
1999–2008	86.8	(80.4–91.2)*	Not attained		88.0	(81.3–92.4)*
2009–2018	85.6	(79.3–90.1)*	Not attained		86.6	(80.1–91.1)*
Medulloblastoma	67.0	(60.0–73.0)	9.8	(7.6–13.7)	67.3	(60.3–73.4)
1989–1998	64.4	(51.7–74.5)	8.1	(5.8–14.6)	64.6	(51.9–74.8)
1999–2008	70.7	(59.0–79.6)	11.1	(6.6–14.6)	71.1	(59.3–80.0)
2009–2018	65.1	(51.0–76.0)	Not attained		65.5	(51.4–76.4)
Intracranial germ cell tumor	90.0	(82.7–94.3)	Not attained		90.6	(83.1–94.9)
Germinoma	93.9	(85.9–97.4)	Not attained		94.3	(86.0–97.7)
1989–1998	95.8	(73.9–99.4)	Not attained		96.2	(71.8–99.5)
1999–2008	89.7	(71.3–96.5)	Not attained		90.0	(71.1–96.8)
2009–2018	96.7	(78.6–99.5)	Not attained		97.1	(75.5–99.7)
Nongerminoma	79.0	(59.1–90.0)	Not attained		80.4	(59.4–91.2)
1989–1998	75.0	(12.8–96.1)	17.6	(1.0–)	79.1	(7.8–97.9)
1999–2008	71.4	(40.6–88.2)	Not attained		73.1	(40.9–89.6)
2009–2018	90.0	(47.3–98.5)	Not attained	(24.4–)	89.8	(44.2–98.6)

*P < .05.

### Pilocytic Astrocytoma

The 454 patients diagnosed with a pilocytic astrocytoma in adult patients had a median age of 31 years (IQR 22–47 years; Table 1) and a male–female ratio of 1.38:1. The incidence rate remained stable over the time period 1999–2018 (EAPC 0.1%, *P* = .23; Figure 1A). The majority of patients underwent a resection (82.6%; Table 1 and Figure 2B), while a minority received radiotherapy as part of primary treatment (9.7%). The resection rate increased from 68.9% during the period 1989–1993 to 88.9% in 1994–1998, after which this remained stable above 80%. No improvement in OS or RS was observed after 1999 (Table 2).

### Medulloblastoma

Two hundred and five cases of adult medulloblastoma were identified, with the classic form—including not otherwise specified subtypes—representing the majority of tumors (75.6%; Table 1), only 4 cases (2.0%) were reported as large cell/anaplastic medulloblastoma. No trend over time was observed for the overall incidence (EAPC −0.5%, *P* = .60; Figure 1A), which remained at around 0.5 per 100 000 inhabitants. In primary treatment, the proportion of patients that received both radiation and chemotherapy increased over time, from 14.7% in 1989–1993 to 50.0% in 2014–2018 (*P* < .01; Figure 2C), while the proportion treated with radiation in the absence of chemotherapy decreased from 76.5% to 40.6%. Although survival appeared better for patients diagnosed during the intermediate time period 1999–2008—with 5-year OS and RS at 70.7% (95% CI: 59.0%–79.6%; Table 2) and 71.1% (95% CI: 59.3%–80.0%), respectively—no significant trend was observed over the total study period.

### Intracranial Germ Cell Tumors

Of the 112 patients diagnosed with primary intracranial germ cell tumors retrieved from the NCR database, germinomas comprised almost 3 quarters (74.1%; Table 1) with a high male predominance (81.9%); among nongerminomas (25.9%), teratomas represented the most frequent group. The majority of germ cell tumors involved or were found near the pineal and/or suprasellar region (37.5% and 17.0%, respectively), while 11.6% were located in the ventricles, and 9.8% in overlapping regions of the CNS (data not shown). The total incidence showed an increasing albeit not statistically significant trend over time (EAPC 2.5%, *P* = .10; Figure 1A). Radiation therapy comprised the mainstay of primary treatment of germinomas (Figure 2D). From 1994–1998 to 2004–2008, an increasing proportion of patients was treated with a combination of radiation therapy and chemotherapy (from 14.3% to 33.3%), after which this appeared to drop to just over 7% in 2014–2018. For nongerminomatous tumors other than teratomas, combined radiation and chemotherapy was added to the treatment arsenal from 1999 onwards (Figure 2E). Patient survival seemed to fluctuate over time: for both germinomas and nongerminomas, 5-year rates (OS and RS) were lower during the intermediate period 1999–2008.

## Discussion

Together, ependymomas, pilocytic astrocytomas, medulloblastomas, and intracranial germ cell tumors constitute a mere 3%–4% of all newly diagnosed and pathologically verified primary CNS neoplasms among adults. This is the first study to provide a comprehensive overview of incident diagnoses of these tumors in the Dutch population, of treatment regimens, and of patients’ prognosis following diagnosis. Overall, the findings of our survey appear to corroborate some of the previous accounts on tumor incidence and patient survival.^4,6,12,17–19^ A recent update from the Central Brain Tumor Registry of the United States (CBTRUS) reported annual incidence rates over 2014–2018 for ependymomas (0.53 per 100 000 inhabitants), pilocytic astrocytomas (0.08 per 100 000), medulloblastomas (0.02 per 100 000), and germ cell tumors (0.01 per 100 000) in patients aged 40 years and over.^10^ Disparities between these and our rates may be related to the different age cutoffs, but differences in (completeness of) case notifications cannot be precluded.

Several issues inherent to population-based observational studies should be considered when interpreting the results. Firstly, the accuracy of diagnoses included in the study constitutes an important concern. Although the survey was conducted using a near-complete registry as of 1999—with incomplete case notification of CNS neoplasms to the NCR only being presumed for histologically unverified benign or low-grade tumors for which patients did not receive treatment and for which they were not admitted to hospital—misdiagnoses should be expected in the absence of a central pathology review. For instance, several studies have reported on misdiagnoses with respect to ependymomas in adults. While around one-tenth of cases in study populations were mentioned to have been reclassified as ependymoma following an initial nonependymoma diagnosis,^20,21^ one study found over 40% of intracranial ependymomas sent in for central review to be other entities, including glioblastomas and oligodendrogliomas.^22^

Secondly, alongside actual changes in the epidemiology and clinical management of the patient groups under study, observed trends need to be appraised against the background of tumor classifications that change over time, as data were collected under multiple different WHO classification schemes.^1,23–26^ In the wake of this, registry protocols have undergone major revisions. In the case of grade I ependymomas (subependymomas and myxopapillary ependymomas) the NCR did not systematically receive case notifications of nonmalignant CNS tumors prior to the introduction of the third edition of the ICD-O.^27^ This is not necessarily true for pilocytic astrocytomas, however, since these became downgraded from malignant to uncertain behavior with ICD-O-3. Nevertheless, analyses on incidence rates and trends involving these tumors were restricted to the years since 1999, but it remains unclear to what extent potential bias has been minimized.

At the same time, trends may also—at least in part—be due to actual changes in tumor incidence. The 30-year study period saw considerable additions to the diagnostic arsenal for CNS neoplasms, including novel applications of neuroimaging techniques and introduction of molecular pathology. Indeed, a decrease in the incidence of unspecified CNS neoplasms was reported earlier for the Netherlands.^28^ In this study, the increase in myxopapillary ependymomas seems to be in line with a rising proportion of resections for ependymal tumors in the spine. Subsequently, a larger proportion of grade I tumors most likely translated to an improved survival for the total patient group over time, although the impact of second-look surgery and overall better postsurgical management should also be taken into account.^29,30^

As the study, data were drawn from a nonselected patient population, they may generally be conceived as reflecting clinical practice over the investigated period. Even though not all diagnoses may have been accurate, they comprised the best information that was available to patients and their treating physicians for making clinical decisions at that time. Aside from the already mentioned increase in surgery for spinal ependymoma, some other treatment patterns are also worth discussing.

Notwithstanding the increased usage over time, a quarter of patients with an anaplastic ependymoma did not receive radiation therapy.^31^ The reasons for this are largely unclear. Perhaps these patients were in too poor condition to undergo radiotherapy, or perhaps a complete resection was anticipated to be sufficient treatment as the evidence on the efficacy of radiation had long remained unconvincing. A more thorough evaluation would anyhow require more detailed data, for instance on patients’ functional status.

In the management of medulloblastomas in adults, the growing proportion of patients receiving chemotherapy, mostly in addition to radiation (craniospinal irradiation), merits consideration. Particularly between 2010 and 2018, the national treatment protocol advised to reserve systemic treatment primarily for high-risk patients only, while after 2018 all medulloblastoma patients were advised adjuvant chemotherapy based on large series and a meta-analysis.^32–34^ The likelihood of a temporal shift in the ratio of high- and low-risk medulloblastomas seems remote as survival did not show to be significantly worse over time. About 1 in 10 patients did not have radiation therapy, which is presumably due to them having comparatively worse performance status or disease stage.

Chemotherapy and radiation (craniospinal or whole ventricle irradiation combined with a local boost) have been mainstays of germinoma management given the tumors’ responsiveness to these modalities. The proportion of patients receiving chemotherapy in the intermediate period 2004–2008 nevertheless appears remarkably high. This observation may be related to initiatives, in children but probably also in adults, to minimize long-term adverse effects by replacing radiation therapy doses and volumes with neoadjuvant or upfront chemotherapy.^35,36^ These initiatives were largely abandoned in clinical practice after chemotherapy only regimens or chemo- combined with focal radiotherapy proved inferior to more extensive irradiation in terms of tumor relapse.^37^

Treatment patterns for nongerminoma germ cell tumors are more difficult to evaluate due to the low number of cases. While patients with teratomas generally only undergo resection of their tumor, chemotherapy and/or radiation were added to the majority of other patients. Indeed, although results obtained in (mostly) pediatric patients suggest that craniospinal radiotherapy could be avoided for localized disease, the combined regimen is regarded as standard management in the metastatic setting.^38^

The observed fluctuations in treatment patterns may be conceived to mirror the changing consensus regarding optimal management of rare CNS tumors over time. In the absence of evidence-based guidelines, the Rare Cancer Working Group of the Dutch Neuro-Oncology Society (LWNO) has since 2011 established and maintained national treatment protocols for the rare tumors discussed in this study (available on www.lwno.nl). Implementation of these protocols aimed to promote more coherent treatment across the Netherlands, thus enabling valid evaluation of treatments. This information should subsequently inform planning of future clinical interventions.

More therapeutic shifts are to be anticipated given the growing understanding of the tumors in this study. Since the introduction of genetic profiles in the fourth version of the WHO classification (2007), molecular analyses supplementing clinicopathologic information have caused important alterations in nearly all tumor classes. DNA methylation profiling, for instance, identified subgroups of ependymal tumors associated with anatomical sites,^39^ and more genetically defined tumors (in addition to supratentorial ependymoma *ZFTA* fusion-positive) have indeed been introduced in the fifth edition of the WHO classification (including supratentorial *YAP1* fusion-positive ependymoma, posterior fossa group A [*PFA*] and *PFB* ependymoma, and spinal ependymoma, *MYCN*-amplified).^40^ In the domain of astrocytomas, a subset of tumors that were often designated as a more aggressive form of pilocytic astrocytomas, has been reclassified as a totally different entity, that is, high-grade astrocytoma with piloid features.^3,9^ While the histopathological classification of medulloblastomas has hitherto been retained given its clinical utility, more distinct, clinically relevant subgroups based on molecular subtype and specific genetic alterations have been defined.^41–43^

In the rapidly altering landscape of CNS tumors, information gathered from comprehensive population-based studies is indispensable for interpreting and understanding the trends observed in the clinic. This is even more true in the case of rare diseases. In addition, results obtained by such registries should confirm the clinical relevance and also chart the consequences of diagnostic and treatment paradigm shifts that are taking place over different time periods.

## Conclusions

Due to their rarity and complex biology, establishing standard treatment protocols for rare CNS tumors is challenging, as is the case for ependymomas, pilocytic astrocytomas, medulloblastomas, and intracranial germ cell tumors in adults. Distilling evidence-based management from scientific studies is further complicated by the significant changes that have taken place and continue to occur in the diagnosis of many of these entities. This study provides information for evaluating such changes, and hence for monitoring patient outcomes for these rare CNS tumors over time, serving as a basis for highly needed treatment protocols.

## Supplementary Material

vdac062_suppl_Supplementary_Table_A

vdac062_suppl_Supplementary_Table_B
