# Supplementary material for: Rare central nervous system tumors in adults: a population-based study of ependymomas, pilocytic astrocytomas, medulloblastomas, and intracranial germ cell tumors
Source: Neurooncol Adv. 2022 Apr 22;4(1):vdac062. doi: 10.1093/noajnl/vdac062 (PMC9154328; doi:10.1093/noajnl/vdac062)
Supplement: vdac062_suppl_Supplementary_Table_A [file vdac062_suppl_supplementary_table_a.docx]

**Table A.** Selection of tumors according to the World Health Organization (WHO) classification of tumors of the central nervous system (4^th^ edition, 2016) and the International Classification of Diseases for Oncology (ICD-O-3) morphology coding

| **Tumor type (WHO 2016)** | **Morphology code (ICD-O-3)** |
| --- | --- |
| **Ependymal tumors** |  |
| Subependymoma | M9383/1 |
| Myxopapillary ependymoma | M9394/1 |
| Ependymoma  Papillary ependymoma  Clear cell ependymoma  Tanycytic ependymoma | M9391/3  M9393/3  M9391/3  M9391/3 |
| Ependymoma, *RELA* fusion-positive | M9396/3 |
| Anaplastic ependymoma | M9392/3 |
| **Pilocytic astrocytoma** |  |
| Pilocytic astrocytoma  Pilomyxoid astrocytoma | M9421/1  M9425/3 |
| **Medulloblastoma** |  |
| Medulloblastomas, genetically defined  Medulloblastoma, WNT-activated  Medulloblastoma, SHH-activated and *TP53*-mutant Medulloblastoma, SHH-activated and *TP53*-wildtype Medulloblastoma, non-WNT/non-SHH | M9475/3  M9476/3  M9471/3  M9477/3 |
| Medulloblastomas, histologically defined  Medulloblastoma, classic  Medulloblastoma, desmoplastic/nodular  Medulloblastoma with extensive nodularity  Medulloblastoma, large cell / anaplastic | M9470/3  M9471/3  M9471/3  M9474/3 |
| Medulloblastomas, NOS | M9470/3 |
| **Intracranial germ cell tumor** |  |
| Germinoma | M9064/3 |
| Embryonal carcinoma | M9070/3 |
| Yolk sac tumor | M9071/3 |
| Choriocarcinoma | M9100/3 |
| Teratoma  Mature teratoma  Immature teratoma | M9080/1  M9080/0  M9080/3 |
| Teratoma with malignant transformation | M9084/3 |
| Mixed germ cell tumor | M9085/3 |
